# Supplementary material for: Recent autumn sea ice loss in the eastern Arctic enhanced by summer Asian-Pacific Oscillation
Source: Nat Commun. 2024 Mar 30;15:2798. doi: 10.1038/s41467-024-47051-8 (PMC10981668; doi:10.1038/s41467-024-47051-8)
Supplement: Supplementary file 1 — Supplementary Information [file 41467_2024_47051_MOESM1_ESM.pdf]

Supplementary Information for:

Recent autumn sea ice loss in the eastern Arctic enhanced by summer Asian-Pacific Oscillation

Botao Zhou[1,2], Ziyi Song[1,2], Zhicong Yin[1,2], Xinping Xu[1,2],

Bo Sun[1,2], Pangchi Hsu[1,2], Haishan Chen[1,2]

[1] Collaborative Innovation Center on Forecast and Evaluation of Meteorological Disasters/Key Laboratory of Meteorological Disaster, Ministry of Education/Joint International Research Laboratory of Climate and Environment Change, Nanjing University of Information Science and Technology, Nanjing, China

[2] School of Atmospheric Sciences, Nanjing University of Information Science and Technology, Nanjing, China

Nature Communications

Corresponding author: Botao Zhou

E-mail: zhoubt@nuist.edu.cn

Supplementary Figures

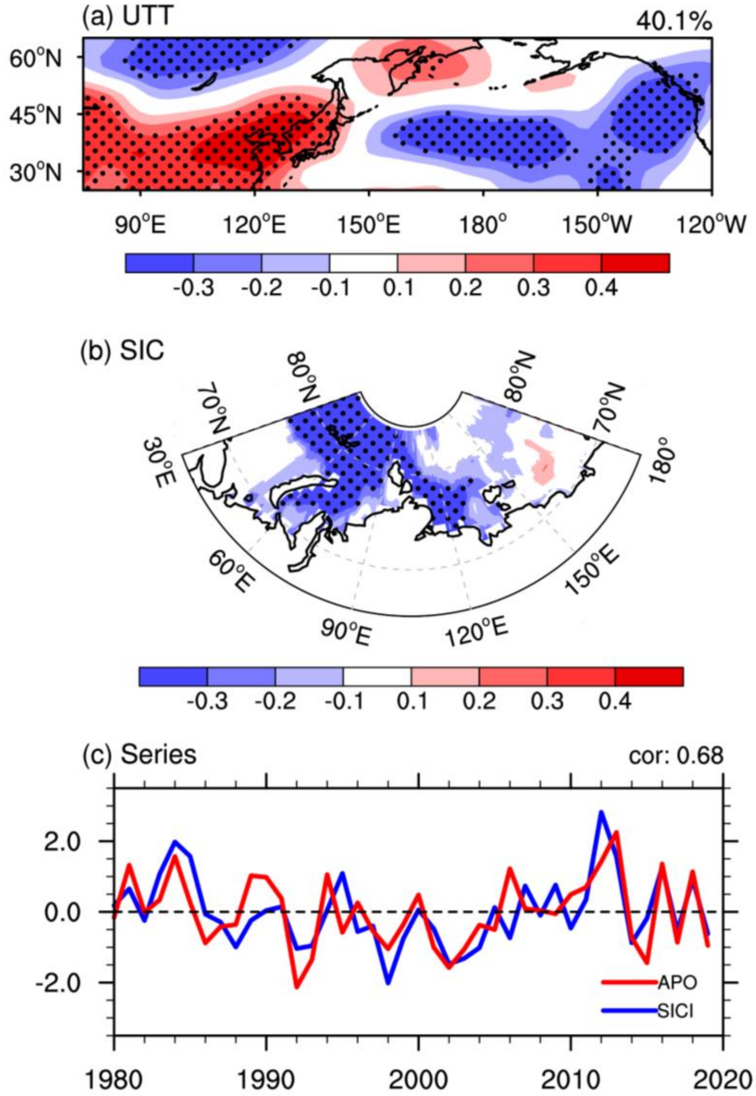

Supplementary Fig. 1 Coupled patterns between summer upper-tropospheric (500-200 hPa) eddy temperature (UTT) over the Asian-Pacific sector and autumn sea ice concentration (SIC) in the eastern Arctic. a–b Spatial patterns of the first leading maximum covariance analysis mode for **a** summer UTT over the Asian-Pacific sector and **b** autumn SIC in the eastern Arctic over the period 1980–2019**.** Areas significant above the 90% confidence level are dotted. **c** Time series of the UTT (red line, defined as the Asian-Pacific Oscillation (APO) index) and SIC (blue line, defined as the SIC index (SICI)) patterns shown in **a** and **b**, respectively. This figure was created by the NCAR Command Language (NCL) Version 6.6.2 (https://www.ncl.ucar.edu/). Source data are provided as a Source Data file.

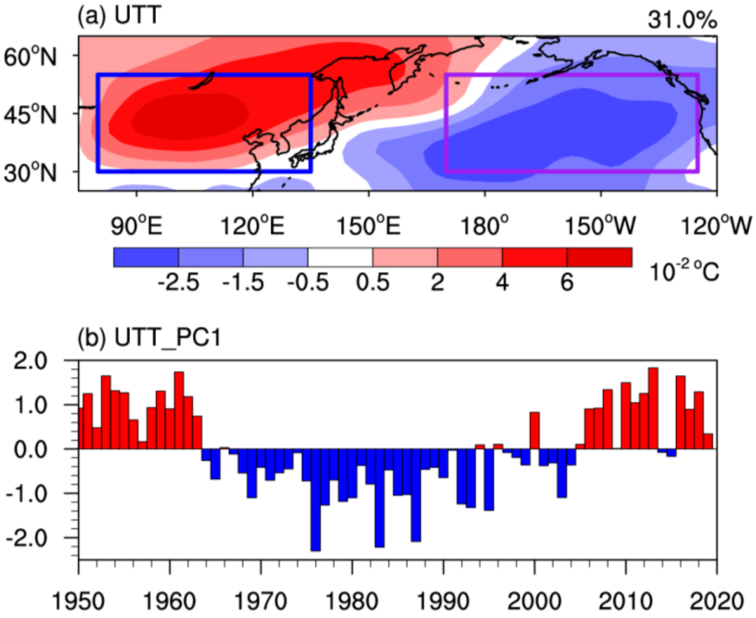

Supplementary Fig. 2 Summer upper-tropospheric (500-200 hPa) eddy temperature (UTT) pattern. a EOF1 mode of summer UTT (10^{-2} ℃) over the Asian-Pacific sector for the period 1950–2019. **b** Normalized time series (PC1; bar) of the EOF1 mode from 1950 to 2019. The blue and purple boxes respectively outline the key regions (30°-55°N, 80°-135°E) and (30°-55°N, 170°E-125°W) adopted to define the Asian-Pacific Oscillation (APO) index for the simulation analysis. This figure was created by the NCAR Command Language (NCL) Version 6.6.2 (https://www.ncl.ucar.edu/). Source data are provided as a Source Data file.

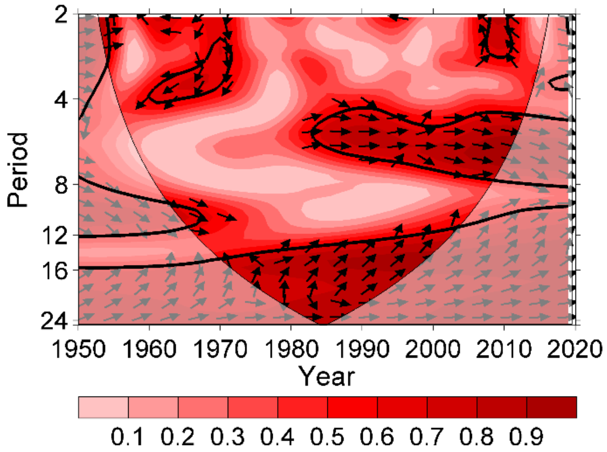

Supplementary Fig. 3 Squared wavelet coherence between summer Asian-Pacific Oscillation (APO) and autumn sea ice concentration indices (SICI). Abscissa is time and ordinate is the period in year. The relative phase relationship is shown as arrows and the 90% confidence level against red noise is shown as thick contours. The parabola indicates the "cone of influence". This figure was created by MATLAB Version R2021B (https://ww2.mathworks.cn/products/matlab.html). Source data are provided as a Source Data file.

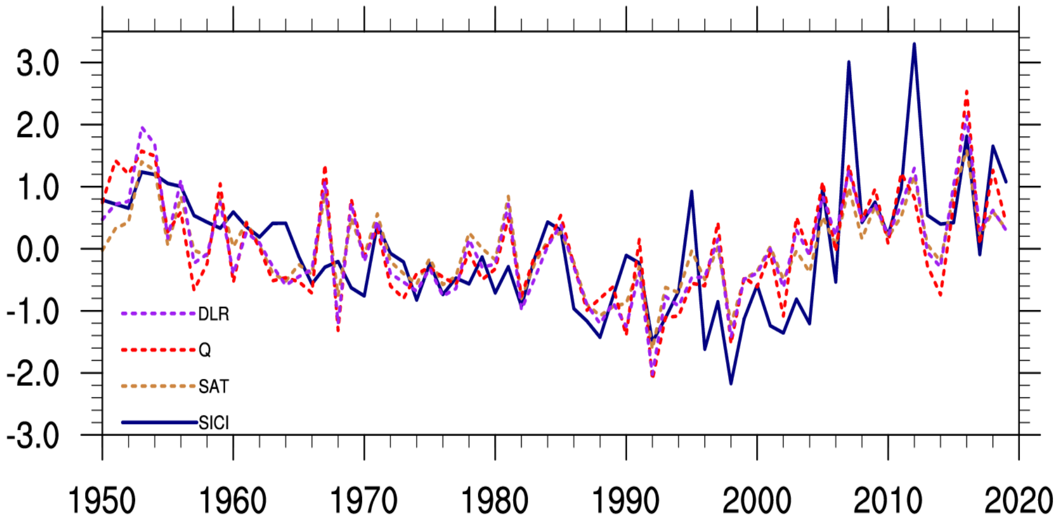

Supplementary Fig. 4 Thermodynamic processes associated with the sea ice concentration index (SICI). Normalized time series of the SICI and the low-level specific humidity (Q), downwelling longwave radiation (DLR), and surface air temperature (SAT) averaged over the eastern Arctic in autumn from 1950 to 2019. This figure was created by the NCAR Command Language (NCL) Version 6.6.2 (https://www.ncl.ucar.edu/). Source data are provided as a Source Data file.

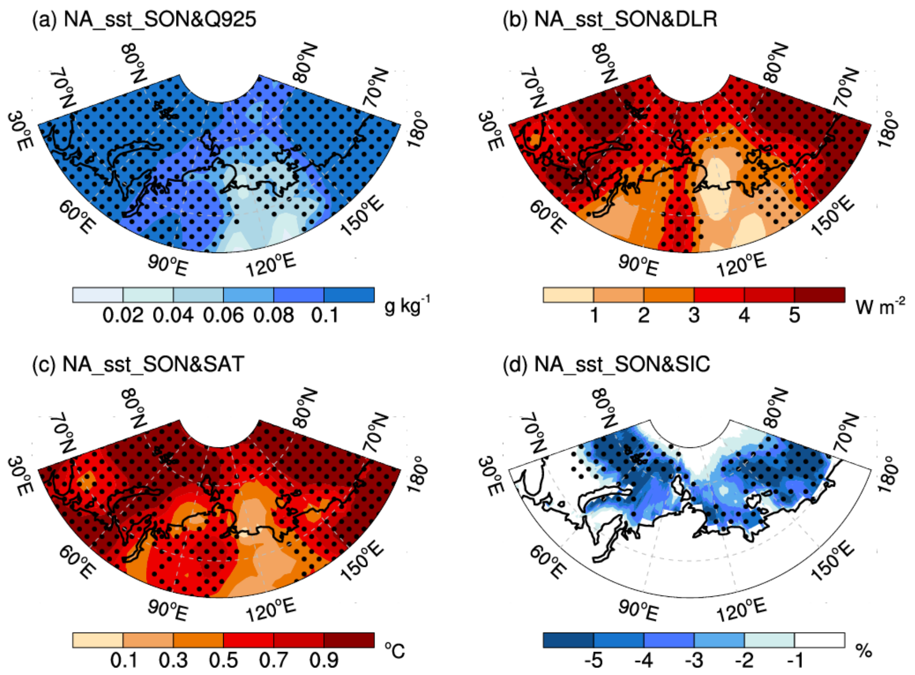

Supplementary Fig. 5 Thermodynamic processes associated with the mid-latitude North Atlantic sea surface temperatures (SSTs) in autumn (September-October-November, SON). a 925 hPa specific humidity (Q925, g kg^{-1}), **b** downwelling longwave radiation (DLR, W m^{-2}), **c** surface air temperature (SAT, °C), and **d** sea ice concentration (SIC, %) regressed onto the normalized NA_sst index (the area-averaged SSTs over the North Atlantic (30 º-50 ºN, 75 º-25 ºW)) over the period 1950–2019. Areas significant above the 90% confidence level are dotted. This figure was created by the NCAR Command Language (NCL) Version 6.6.2 (https://www.ncl.ucar.edu/). Source data are provided as a Source Data file.

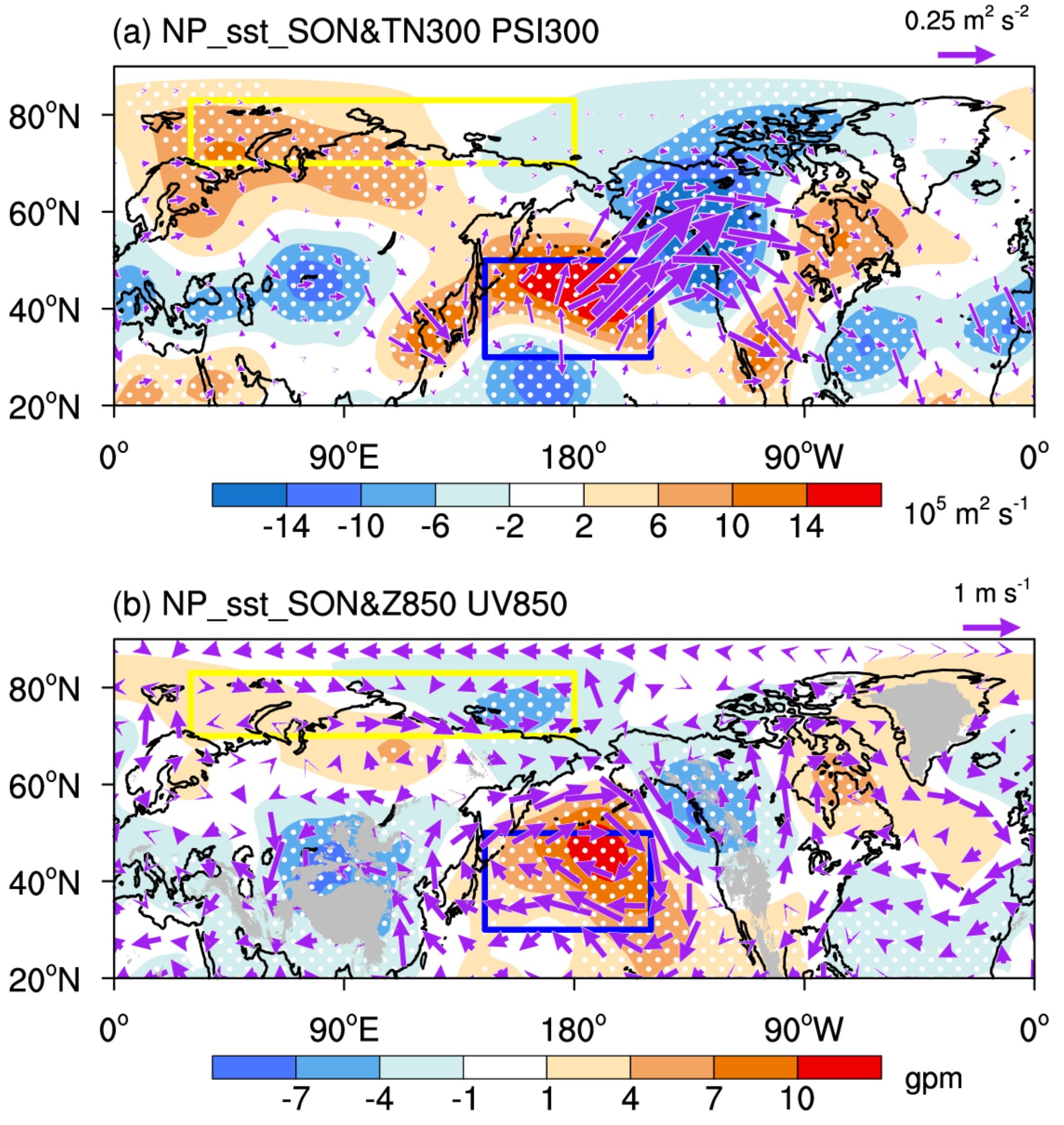

Supplementary Fig. 6 Atmospheric pattern associated with the mid-latitude North Pacific sea surface temperatures (SSTs) in autumn (September-October-November, SON). **a** Horizontal wave activity flux (TN300, vectors, m^2 s^{-2}) and stream function (PSI300, shading, 10^5 m^2 s^{-1}) at 300 hPa in association with the normalized NP_sst index (the area-averaged SSTs over the North Pacific (30 °-50 °N, 145 °E-150 °W)). **b** 850 hPa horizontal winds (UV850, m s^{-1}) and geopotential height (Z850, shading, gpm) regressed onto the normalized NP_sst index. Anomalies significant above the 90% confidence level are dotted. The blue and yellow boxes outline the North Pacific (30 °-50 °N, 145 °E-150 °W) and the eastern Arctic sea ice (70 °-83 °N, 30 °E-180 °) domains. This figure was created by the NCAR Command Language (NCL) Version 6.6.2 (https://www.ncl.ucar.edu/). Source data are provided as a Source Data file.

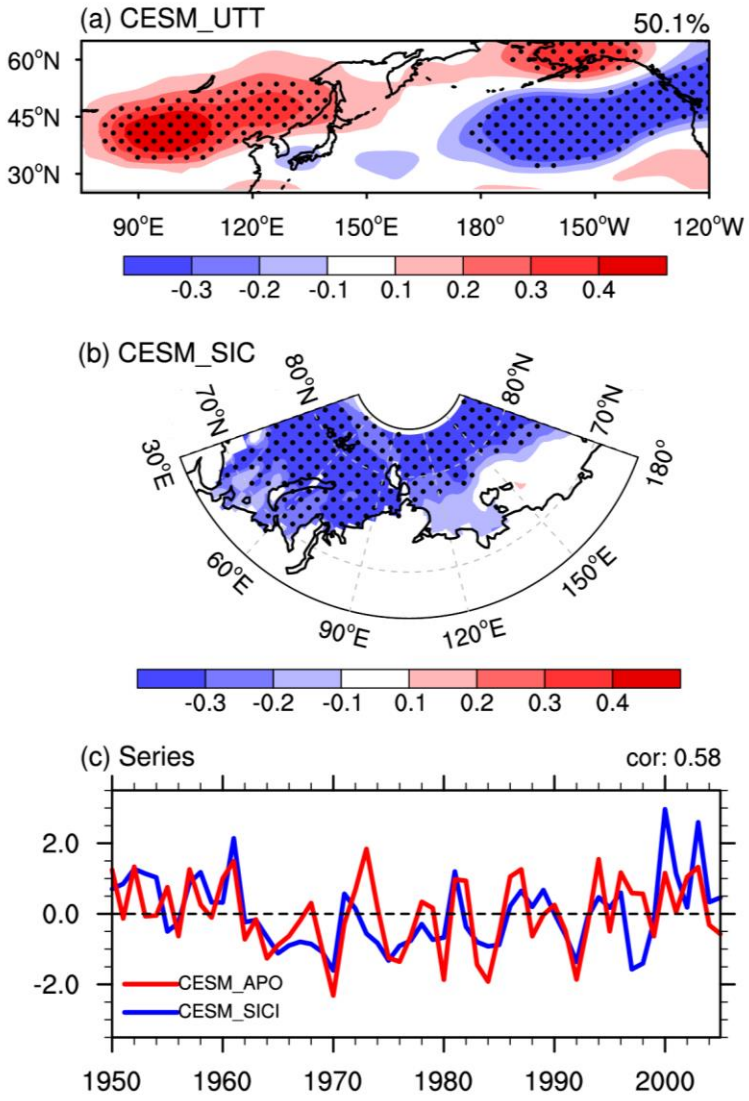

Supplementary Fig. 7 BMME simulated coupled patterns between summer upper-tropospheric (500-200 hPa) eddy temperature (UTT) over the Asian-Pacific sector and autumn sea ice concentration (SIC) in the eastern Arctic. **a**–**b** Spatial patterns of the first leading maximum covariance analysis mode for **a** summer UTT over the Asian Pacific sector and **b** autumn SIC in the eastern Arctic over the period 1950–2005. Areas significant above the 90% confidence level are dotted. **c** Time series of the UTT (red line, defined as the Asian-Pacific Oscillation (APO) index) and SIC (blue line, defined as the SIC index (SICI)) patterns respectively shown in **a** and **b**. This figure was created by the NCAR Command Language (NCL) Version 6.6.2 (https://www.ncl.ucar.edu/). Source data are provided as a Source Data file.

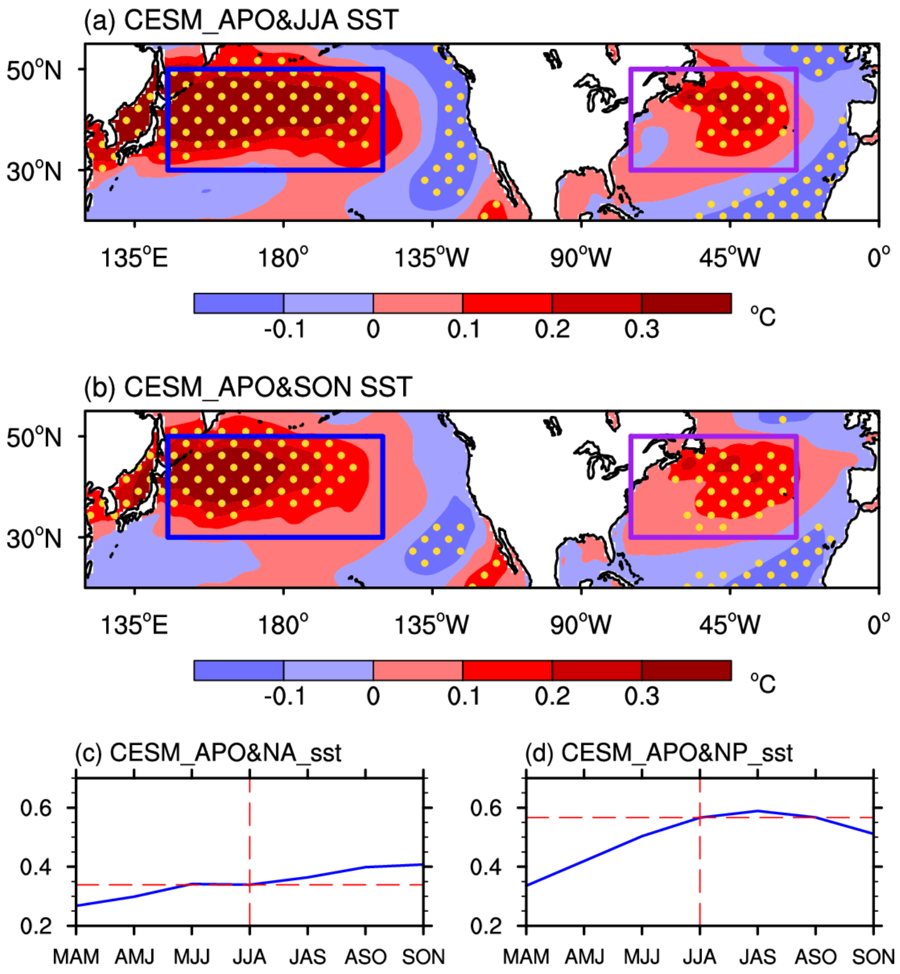

Supplementary Fig. 8 BMME simulated evolution of Asian-Pacific Oscillation (APO)-associated changes in sea surface temperatures (SSTs) from summer to autumn. a summer and **b** autumn SSTs (℃) regressed onto the normalized summer APO index over the period 1950–2005. Areas significant above the 90% confidence level are dotted. The blue and purple boxes in **a** and **b** outline the North Pacific (30°-50°N, 145°E-150°W) and North Atlantic (30°-50°N, 75°-25°W) domains used for the definition of NP_sst and NA_sst, respectively. **c-d** Lead-lag correlations of the summer APO index with **c** the NA_sst and **d** the NP_sst. The dashed vertical lines in **c** **(d)** represents the simultaneous correlation of APO with the NA_sst (NP_sst) in June-July-August (JJA), to the left of which indicates the NA_sst (NP_sst) leading the APO and to the right of which indicates the APO leading the NA_sst (NP_sst). MAM, AMJ, MJJ, JJA, JAS, ASO, SON indicate the March-April-May, April-May-June, May-June-July, June-July-August, July-August-September, August-September-October, and September-October-November, respectively. This figure was created by the NCAR Command Language (NCL) Version 6.6.2 (https://www.ncl.ucar.edu/). Source data are provided as a Source Data file.

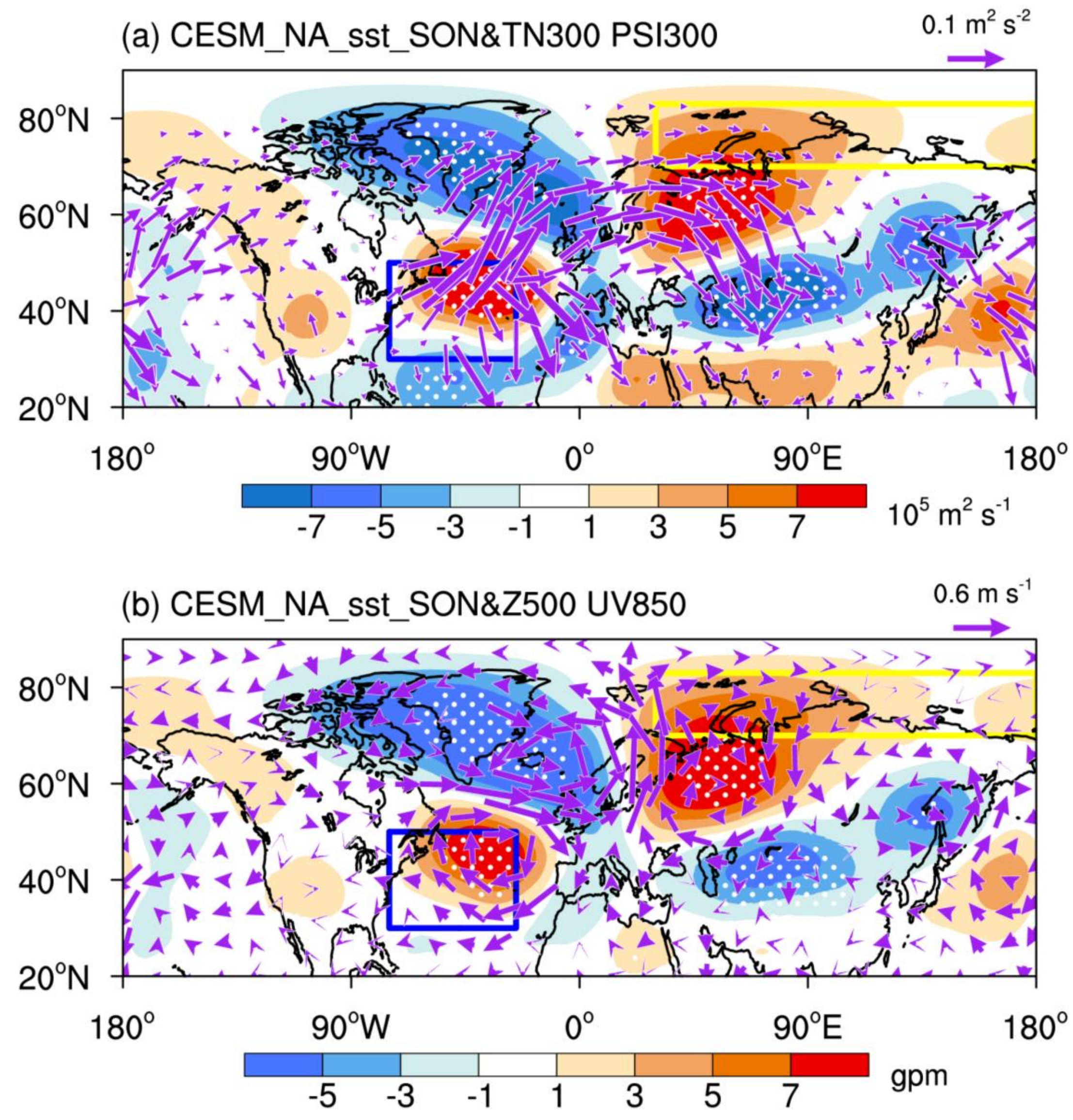

Supplementary Fig. 9 BMME simulated atmospheric pattern associated with the mid-latitude North Atlantic sea surface temperatures (SSTs) in autumn (September-October-November, SON). **a** Horizontal wave activity flux (TN300, vectors, m^2 s^{-2}) and stream function (PSI300, shading, 10^5 m^2 s^{-1}) at 300 hPa in association with the normalized NA_sst index (the area-averaged SSTs over the North Atlantic (30°-50°N, 75°-25°W)). **b** 850 hPa horizontal winds (UV850, m s^{-1}) and 500 hPa geopotential height (Z500, shading, gpm) regressed onto the normalized NA_sst index. Areas significant above the 90% confidence level are dotted. The blue and yellow boxes outline the North Atlantic (30°-50°N, 75°-25°W) and the eastern Arctic sea ice (70°-83°N, 30°E-180°) domains, respectively. This figure was created by the NCAR Command Language (NCL) Version 6.6.2 (https://www.ncl.ucar.edu/). Source data are provided as a Source Data file.

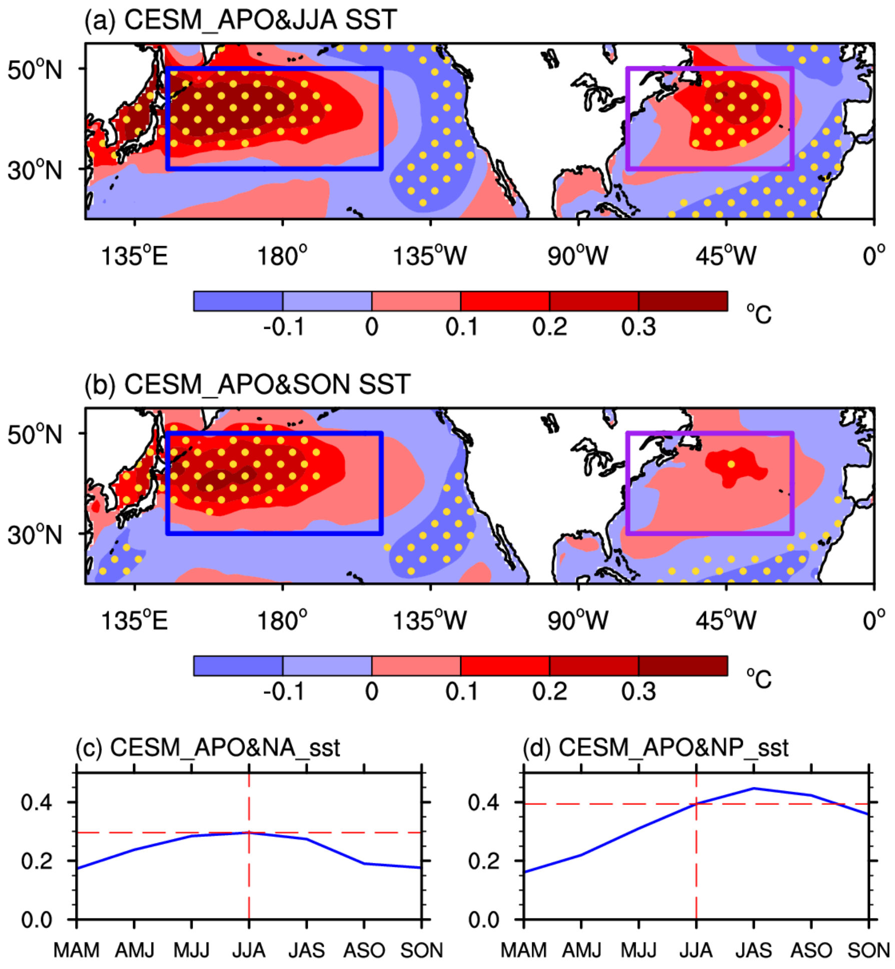

Supplementary Fig. 10 WMME simulated evolution of Asian-Pacific Oscillation (APO)-associated changes in sea surface temperatures (SSTs) from summer to autumn. **a** summer and **b** autumn SSTs (℃) regressed onto the normalized summer APO index over the period 1950–2005. Areas significant above the 90% confidence level are dotted. The blue and purple boxes in **a** and **b** outline the North Pacific (30°-50°N, 145°E-150°W) and North Atlantic (30°-50°N, 75°-25°W) domains used for the definition of NP_sst and NA_sst, respectively. **c-d** Lead-lag correlations of the summer APO index with **c** the NA_sst and **d** the NP_sst. The dashed vertical lines in **c** **(d)** represents the simultaneous correlation of APO with the NA_sst (NP_sst) in June-July-August (JJA), to the left of which indicates the NA_sst (NP_sst) leading the APO and to the right of which indicates the APO leading the NA_sst (NP_sst). MAM, AMJ, MJJ, JJA, JAS, ASO, SON indicate the March-April-May, April-May-June, May-June-July, June-July-August, July-August-September, August-September-October, and September-October-November, respectively. This figure was created by the NCAR Command Language (NCL) Version 6.6.2 (https://www.ncl.ucar.edu/). Source data are provided as a Source Data file.

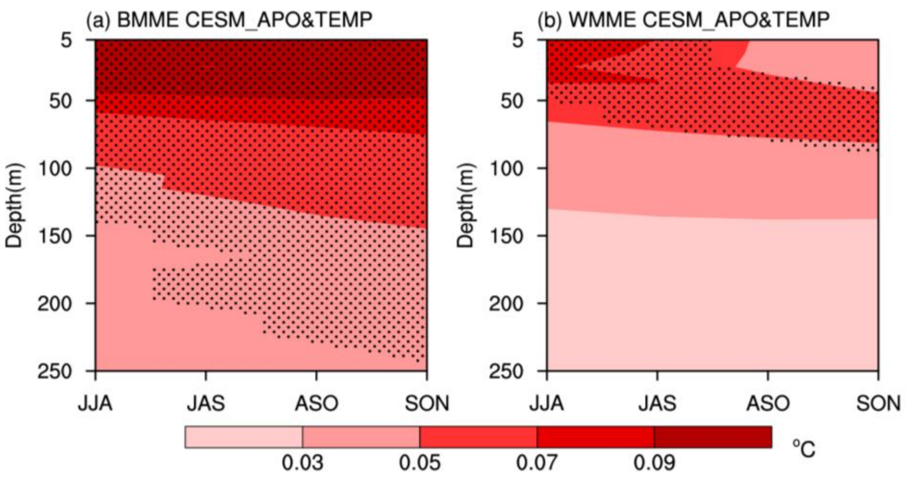

Supplementary Fig. 11 Simulated evolution of Asian-Pacific Oscillation (APO)-associated changes in oceanic temperatures in the North Atlantic from summer to autumn. a BMME and **b** WMME simulated area-averaged oceanic temperatures (TEMP, ℃) over the North Atlantic (30 º-50 ºN, 75 º-25 ºW) regressed onto the normalized summer APO index over the period 1950–2005. Areas significant above the 90% confidence level are dotted. This figure was created by the NCAR Command Language (NCL) Version 6.6.2 (https://www.ncl.ucar.edu/). Source data are provided as a Source Data file.
