# Supplementary material for: Recent autumn sea ice loss in the eastern Arctic enhanced by summer Asian-Pacific Oscillation
Source: Nat Commun. 2024 Mar 30;15:2798. doi: 10.1038/s41467-024-47051-8 (PMC10981668; doi:10.1038/s41467-024-47051-8)
Supplement: Supplementary file 3 — Inventory of Supplementary Information [file 41467_2024_47051_MOESM3_ESM.docx]

**Supplementary Information for:**

**Recent autumn sea ice loss in the eastern Arctic enhanced by s****ummer Asian-Pacific Oscillation**

Botao Zhou^1,2^, Ziyi Song^1,2^, Zhicong Yin^1,2^, Xinping Xu^1,2^,

Bo Sun^1,2^, Pangchi Hsu^1,2^, Haishan Chen^1,2^

^1^ Collaborative Innovation Center on Forecast and Evaluation of Meteorological Disasters/Key Laboratory of Meteorological Disaster, Ministry of Education/Joint International Research Laboratory of Climate and Environment Change, Nanjing University of Information Science and Technology, Nanjing, China

^2^ School of Atmospheric Sciences, Nanjing University of Information Science and Technology, Nanjing, China

*Nature Communications*

**Corresponding author:** Botao Zhou

E-mail: [zhoubt@nuist.edu.cn](file:///C:\Users\zhoubt\Desktop\待处理\文章待改\宋子袆\1\正式编写稿\bianxie\新图202205\再写202212-待开始\zhoubt@nuist.edu.cn)

**Supplementary information includes**

**Supplementary Figures: Supplementary Figs. 1-11**
